# Retrograde gastrostomy-assisted recanalization for refractory post-caustic esophageal stricture

**DOI:** 10.1055/a-2687-6742

**Published:** 2025-09-05

**Authors:** Jingjing Yao, Guangrong Wang, Wen Jiao, Hongyuan Cui, Jindong Fu

**Affiliations:** 1 Department of Gastroenterology, Rizhao People’s Hospital, Rizhao, China; 2 372527 Shandong Second Medical University, Weifang, China; 3 654581 Jining Medical University Clinical Medical College, Jining, China

A 44-year-old man suffered from a long-segment esophageal stricture after accidental caustic alkali ingestion 2 years earlier. Despite repeated endoscopic esophageal dilations at 1–2-week intervals, he continued to experience persistent dysphagia. To improve his nutritional status, percutaneous endoscopic gastrostomy (PEG) was performed 2 months ago. As the patient insisted on resuming oral intake, another attempt at endoscopic therapy was undertaken.

Conventional anterograde endoscopy revealed extensive circumferential scarring. At 28 cm from the incisors, the esophageal lumen was nearly obliterated, preventing guidewire passage ( Fig. 1 **a** ). After obtaining informed consent, an endoscopic stenosis incision was attempted. A longitudinal radial incision was made, extending approximately 5 cm, but the esophageal lumen remained inaccessible, and a suspected perforation was detected ( Fig. 1 **b** ). We therefore switched to a retrograde strategy ( Video 1 ).

**Fig. 1:**
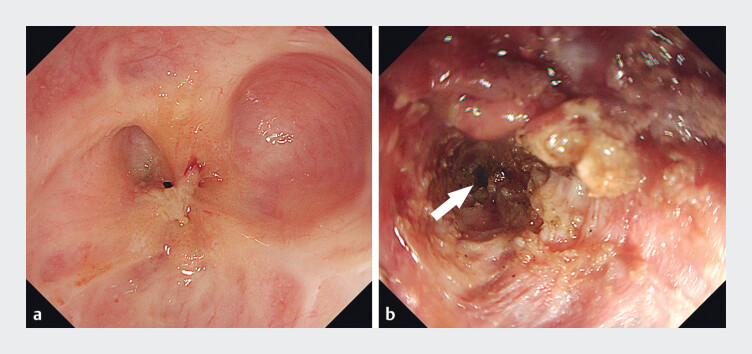
Endoscopic images. **a** At 28 cm from the incisors, the esophageal lumen was almost completely obliterated. **b** After longitudinal incision, a suspected perforation was detected (white arrow) and the lumen remained impassable.

Retrograde gastrostomy-assisted recanalization of a refractory post-caustic esophageal stricture.Video 1

Via the mature PEG tract, the gastroscope was introduced into the stomach and advanced retrogradely through the cardia. Dense fibrosis was encountered in the distal esophagus. Further retrograde advancement revealed a severely narrowed lumen with two small openings ( Fig. 2 ), one of which had a blind ending, while the other permitted the guidewire to pass through with minimal resistance. Upon re-entering the esophagus orally, the guidewire was visualized emerging from the incision ( Fig. 3 ), grasped with biopsy forceps, and externalized through the mouth. Under fluoroscopic guidance, a 10-cm-long fully covered self-expandable metal stent was placed along the guidewire and subsequently deployed ( Fig. 4 ). Reinsertion of the endoscope orally confirmed optimal stent positioning ( Fig. 5 ). The stent was maintained for 4 weeks before removal. The patient was informed that he would require intermittent endoscopic dilations in the future.

**Fig. 2:**
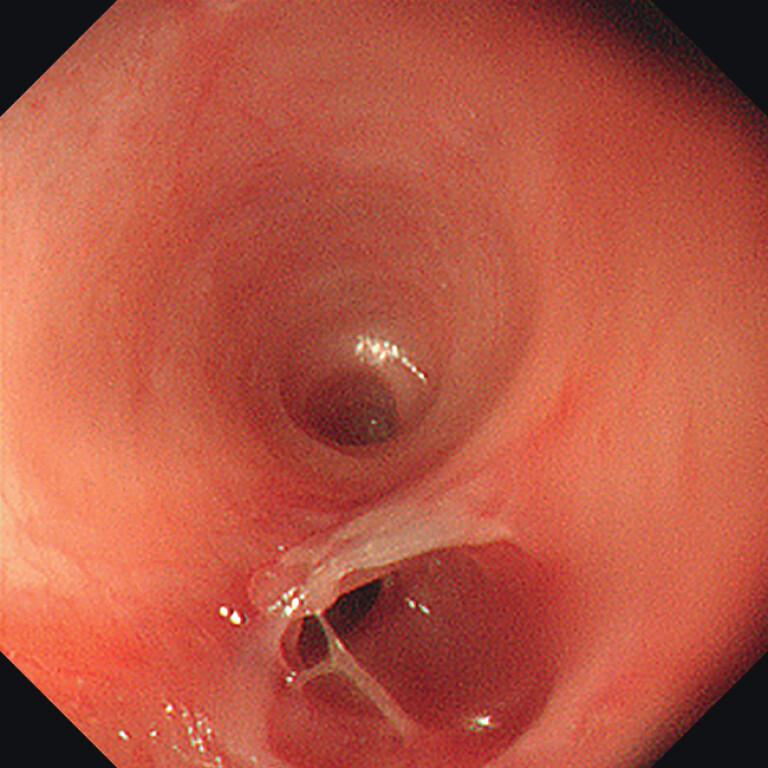
Retrograde view revealed a severely narrowed lumen with two small openings.

**Fig. 3:**
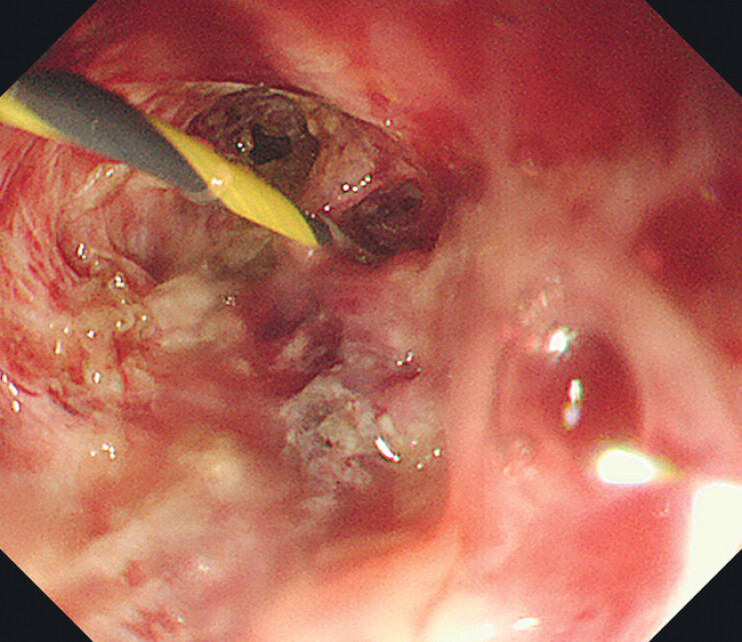
The guidewire was visualized emerging from the incision.

**Fig. 4:**
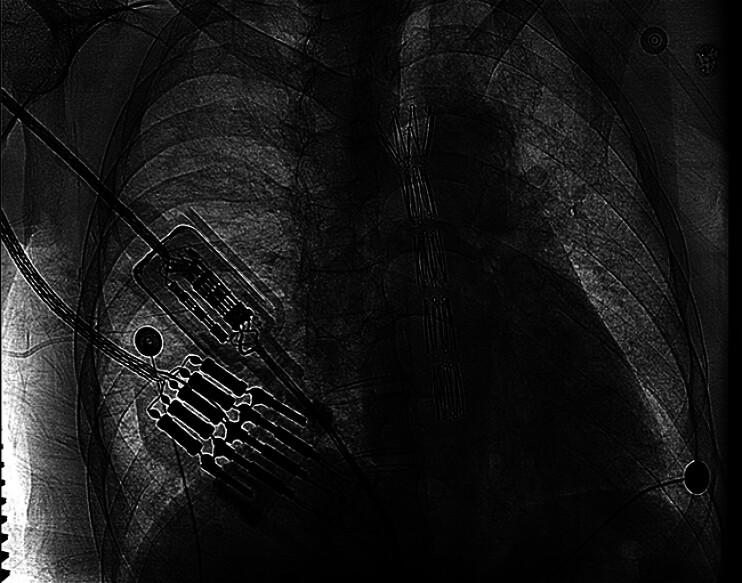
Fluoroscopic confirmation of a 10-cm fully covered self-expandable metal stent deployed across the stricture.

**Fig. 5:**
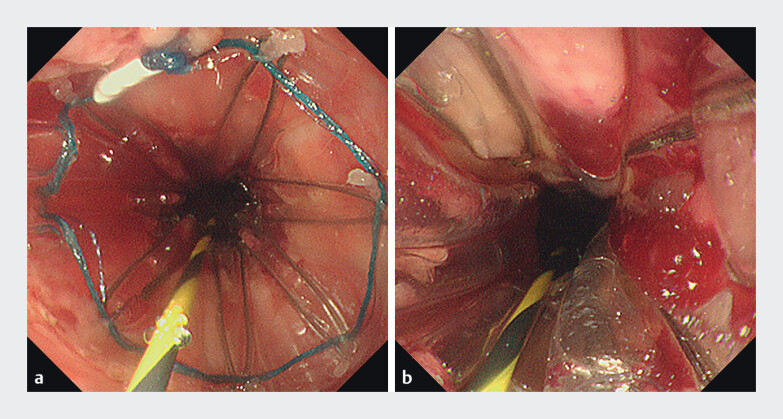
Endoscopic view showing optimal stent placement and full expansion.

Benign esophageal strictures following caustic injury often demand iterative endoscopic therapy and carry substantial perforation risk 1 . This case illustrates that, when the anterograde route fails or is unsafe, retrograde access through an existing gastrostomy tract offers a practical, minimally invasive rescue pathway for complete esophageal recanalization.

Endoscopy_UCTN_Code_TTT_1AO_2AH

## Notes

Endoscopy E-Videos https://eref.thieme.de/e-videos
*E-Videos* is an open access online section of the journal *Endoscopy* , reporting on interesting cases and new techniques in gastroenterological endoscopy. All papers include a high-quality video and are published with a Creative Commons CC-BY license. Endoscopy E-Videos qualify for HINARI discounts and waivers and eligibility is automatically checked during the submission process. We grant 100% waivers to articles whose corresponding authors are based in Group A countries and 50% waivers to those who are based in Group B countries as classified by Research4Life (see: https://www.research4life.org/access/eligibility/ ). This section has its own submission website at https://mc.manuscriptcentral.com/e-videos .
